# L-shaped correlation between serum alpha-1-acid glycoprotein concentration and urinary albumin creatinine ratio in females: a cross-sectional survey

**DOI:** 10.3389/fendo.2025.1438695

**Published:** 2025-03-24

**Authors:** Junjie Wang, Li Xiao, Yuxuan Zhang, Zhou Li

**Affiliations:** ^1^ Department of Geriatrics, First Affiliated Hospital, Army Medical University, Chongqing, China; ^2^ School of Health Management Policy, Peking Union Medical College, Chinese Academy of Medical Sciences, Beijing, China; ^3^ Department of Clinical Laboratory Medicine, Southwest Hospital, Third Military Medical University (Army Medical University), Chongqing, China

**Keywords:** alpha-1-acid glycoprotein, urinary albumin-creatinine ratio, l-shaped correlation, threshold, kidney disease, NHANES

## Abstract

**Background:**

Alpha-1-acid glycoprotein (AGP) is a vital acute phase reactant that increases when glomerular filtration is impaired, making it a potential biomarker of kidney disease. The urine albumin-to-creatinine ratio (UACR) is a sensitive indicator of proteinuria and is frequently used to screen for kidney disease in its early stage. The aim of this study was to explore their correlation in order to advance our understanding of the mechanisms underlying kidney damage.

**Methods:**

This study included 2579 female participants with serum AGP and UACR from the National Health and Nutrition Examination Survey (2015-2018). We divided all participants equally into three groups based on their serum AGP concentration. The univariate and multivariate regression models were for assessing the correlation between AGP and UACR. Subgroup analyses were then performed to explore the effect of each covariate on the correlation. Smoothing splines was utilized to explore their nonlinear correlation and identify thresholds within it.

**Results:**

After adjusting for multivariate models, AGP was significantly and positively associated with UACR (p<0.0001). The study identified a specific cohort of non-Hispanic Black individuals under 20 years of age, characterized by a BMI below 25 kg/m² and a waist circumference of 80 cm or more. Within this cohort, those with hypertension and sleep disorders but without hypercholesterolemia or diabetes exhibited significantly higher UACR (p < 0.001). Furthermore, we discovered an L-shaped correlation between serum AGP concentration and UACR. Specifically, when the serum AGP concentration was less than 140 mg/dL, the UACR plateaued.

**Conclusions:**

This study is the first to address the correlation between serum AGP and UACR and found an L-shaped correlation with a threshold of 140 mg/dl. This could be a target for intervention to reduce the risk of kidney disease.

## Introduction

Serum alpha-1 acid glycoprotein (AGP) concentration serves as a crucial biomarker in various clinical situations due to its acute-phase response in inflammation, infection and other pathological conditions (1, 2). AGP, which is produced mainly in the liver, plays multifaceted roles in modulating immune responses, drug binding and transport^2^. Its levels are influenced by various factors, including age, gender, genetics and hormonal fluctuations (1–3). Clinically, elevated AGP levels are associated with inflammatory diseases such as rheumatoid arthritis, sepsis and certain cancers, while decreased levels may indicate liver dysfunction or malnutrition (4–6). The measurement of serum AGP concentration aids in diagnosing and monitoring the progression of inflammatory diseases, guiding therapeutic interventions, and assessing the severity of illnesses (3). Furthermore, AGP has garnered interest as a potential prognostic marker for predicting patient outcomes and response to treatment (3, 7). Thus, understanding the dynamics of serum AGP concentration is pivotal for optimizing clinical management strategies and enhancing patient care in various medical fields.

The ratio of albumin in urine to the corresponding creatinine concentration, commonly referred to as urinary albumin-creatinine ratio (UACR), serves as a crucial indicator for assessing kidney health and detecting kidney abnormalities (8). Albuminuria, the presence of high levels of urinary albumin, is an early sign of kidney damage or dysfunction. It is commonly associated with conditions such as diabetic nephropathy, hypertension-induced kidney injury, and glomerulonephritis (8–10). Creatinine, a waste product of muscle metabolism, is excreted into the urine at a relatively constant rate and represents a stable marker for urine concentration (11). By normalizing albumin levels to creatinine concentration, the UACR accounts for fluctuations in urine dilution, ensuring a more accurate assessment of albumin excretion. Elevated UACR values indicate increased renal permeability and signify heightened risk for progressive kidney disease, cardiovascular complications, and mortality (8). Therefore, regular monitoring of UACR is fundamental in the early detection, management and prognosis of kidney diseases. In clinical practice, UACR measurements are routinely utilized for screening individuals at risk of kidney dysfunction, monitoring disease progression and assessing the effectiveness of therapeutic interventions, such as angiotensin-converting enzyme inhibitors or angiotensin receptor blockers (8, 12), which are commonly employed to relieve albuminuria and preserve kidney function.

In females, the joint assessment of UACR and AGP may offer a comprehensive view of renal function and inflammation. Research indicates that in autoimmune diseases such as systemic lupus erythematosus (SLE), elevated AGP levels are indicative of systemic inflammation, while increased UACR values suggest kidney - related problems (13). During pregnancy, by monitoring AGP as an inflammatory marker and UACR as a kidney function marker, it is possible to identify women at risk of developing preeclampsia or gestational diabetes (14). For chronic conditions like diabetes, the combined evaluation of AGP and UACR benefits female patients (15). It enables early detection of kidney injury and helps formulate individualized treatment plans, thus enhancing overall disease management.

However, the correlation between them remains unclear at present. Some literatures indicated that when inflammation occurs, pro-inflammatory cytokines will prompt the liver to produce more AGP. At the same time, the inflammatory state will damage the glomerular filtration barrier of the kidneys (16). As more albumin leaks into the urine, the UACR will also increase. AGP, which has anti-inflammatory properties, can regulate CD163 in macrophages, causing them to transform into the anti-inflammatory M2 type (17). This will alleviate renal inflammation, reduce glomerular damage, and potentially decrease the UACR. Conversely, a high UACR indicates severe renal damage, which will disrupt normal renal function. The resulting accumulation of metabolic waste products and protein loss may affect the production and function of AGP in the liver, thus forming a complex feedback loop between these two markers (2, 19, 20). Therefore, revealing the correlation between them is of great urgency. In this study, the aim was to explore the correlation between AGP and UACR in females and to evaluate their combined potential as biomarkers for early kidney disease detection.

## Methods

### Study design and participants

**Figure 1:**
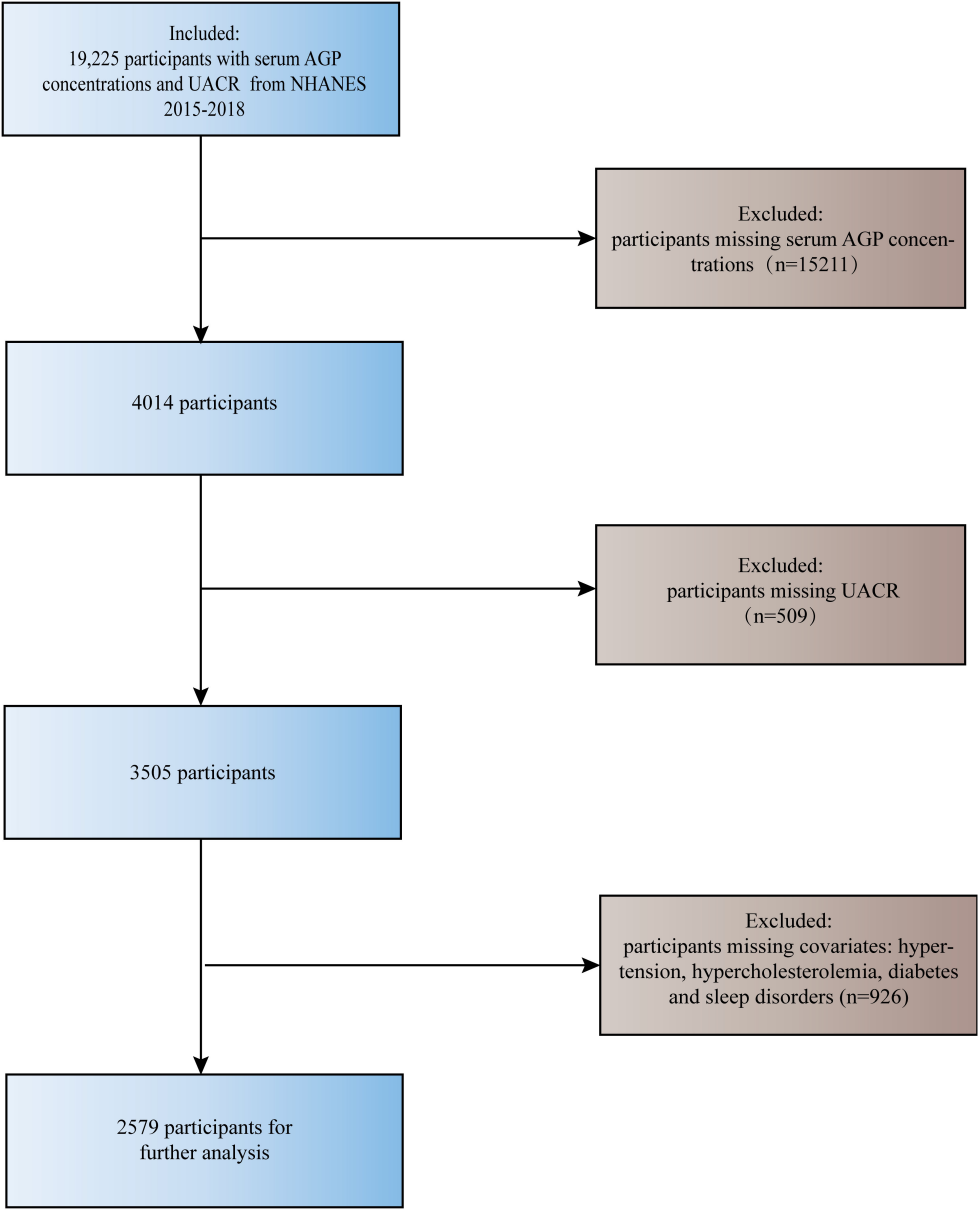
Flowchart for participant screening.

The National Health and Nutrition Examination Survey (NHANES) is an extensive and well-documented program launched by the Centers for Disease Control (CDC) to study Americans. It contains a multitude of data on physical examinations, laboratory tests, questionnaires, and more. The protocols for this program are approved by the Research Ethics Review Board of NCHS. Access to the datasets utilized in this study is available through the NHANES website (https://www.cdc.gov/nchs/nhanes/index.htm) (18). We downloaded datasets with serum AGP concentrations and UACR from NHANES (2015-2016, 2017-2018). This dataset consists of information on the demographic characteristics and disease history of 19,225 participants. Then, a total of 2579 participants were included in this study for further analysis, after excluding missing serum AGP concentrations (N=15211), missing UACR (N=509), and missing the history of hypertension, hypercholesterolemia, diabetes, and sleep disorders (N=926) (**Figure 1**). This study included Caucasians, African-Americans, Hispanics, and all other races in the U.S. and was not designed for a specific ethnic group. Interestingly, after data cleaning, the only participants left in this study were females.

### Measurement of serum AGP concentrations and urinary albumin to creatinine

Serum AGP concentrations were measured using the Tina-quant Roche AAGP2 assay with the AAGP2 Tina-quant α1-Acid Glycoprotein Gen.2 kit (21). The raw data are reported in g/L, and for consistency, the units used in this study are mg/dl after conversion. Urinary albumin was measured using the fluorescent immunoassay (FIA) (22). Urinary creatinine was measured using an endpoint reaction enzymatic method at 546 nm (with a secondary wavelength of 700 nm). UACR is the ratio of urinary albumin to urinary creatinine in mg/g. Since the methods of measurement of serum AGP concentration and urine albumin-to-creatinine ratio are both recommended by the Clinical and Laboratory Standards Institute (CLSI) and the whole process of measurement is supervised by CLSI, the data obtained in this study have a high degree of reliability, which provides a strong assurance of more reliable results in this study.

### Assessment of covariates

It’s reported that demographic information such as age and gender are the most commonly used covariates (23, 24). In addition, participants’ history of common medical conditions, especially those in which an inflammatory state exists, should also be considered as our covariates (24). On this basis, eight covariates including age, race, Body Mass Index (BMI), waist circumference, history of hypertension, hypercholesterolemia, diabetes, and sleep disorders were enrolled in this study. Age and race were obtained through a questionnaire, while BMI (kg/m²) and waist circumference (cm) were measured during a physical examination. According to the World Health Organization’s recommended guidelines (14), individuals over 20 years old are considered adults. A BMI of 18.5 kg/m^2^ – 24.9 kg/m^2^ indicates normal weight, a BMI between 25 kg/m² and 30 kg/m² indicates overweight, and a BMI over 30 kg/m² indicates obesity. A waist circumference of ≥80 cm implies obesity. The definitions of hypertension, hypercholesterolemia, diabetes, and sleep disorders involve a positive response to the following questions: (1) Have you ever been told by a doctor or other health professional that you had hypertension, also called high blood pressure? (2) Have you ever been told by a doctor or other health professional that your blood cholesterol level was high? (3) Have you ever been told by a doctor or health professional that you have diabetes or other health problems? Participants with hypertension, hypercholesterolemia, and sleep disorder were classified as yes and no, as recommended by the guidelines. Meanwhile, participants with diabetes mellitus were categorized as yes, no, and borderline (19). In order to explore the different inflammatory states that may exist at different serum AGP concentrations, based on their serum AGP concentrations, all participants in this study were equally divided into three groups: Group 1 with AGP less than 63 mg/dl (n=855), Group 2 with AGP in the range of 63-84 mg/dl (n=864) and Group 3 with AGP greater than 84 mg/dl (n=860).

### Statistical analyses

Statistical analyses for this study were primarily conducted using the statistical packages R (version 3.4.4) and Empower (version 2.1). Bilateral p < 0.05 was considered statistically significant. Kruskal Wallis rank sum test was conducted for continuous variables, while Fisher’s exact probability test was used for categorical variables with theoretical numbers less than 10 to analyze the characteristics of the participant groups.

Three Cox regression models were designed to examine the correlation between serum AGP concentrations and UACR: model 1 (unadjusted); model 2 adjusted for age and race; and model 3 adjusted for age, race, BMI, waist circumference, history of hypertension, hypercholesterolemia, diabetes mellitus, and sleep disorders. However, since multiple testing may increase the risk of Type I errors, we performed multiple sensitivity analyses and employed the Benjamin-Hodgberg procedure to adjust for multiple testing issues.

To investigate the non-linear correlation between serum AGP concentrations and UACR, Cox proportional risk regression models were developed using smoothed curve fitting (penalized spline method) in Model 3. If a nonlinear correlation existed, we utilized a recursive algorithm to calculate the point of infection between serum AGP concentrations and UACR, and then applied a two-piecewise linear regression model to investigate the correlation between serum AGP concentrations and UACR on either side of the point of infection.

Finally, stratified analyses were conducted based on age, race, BMI, waist circumference, history of hypertension, hypercholesterolemia, diabetes, and sleep disorders. We assessed the presence of interaction effects between these variables using interaction terms.

## Results

### Baseline characteristics of participants

A total of 2579 female participants who met the inclusion criteria were included in this study, of which the average age of participants in each group was 29.98 ± 9.85, 31.90 ± 10.19, and 32.90 ± 10.24 years, respectively. There was a significant difference in age among the groups (p < 0.001). The baseline characteristics of participants in each group based on serum AGP concentration are listed in **Table 1**. Participants with higher serum AGP concentrations were more likely to be older and non-Hispanic white, to have a higher BMI, waist circumference, and were less likely to have hypertension, diabetes, and sleep disorders (p < 0.001). The UACR for each group was 18.03 ± 82.63, 38.21 ± 301.63, and 57.35 ± 737.85 mg/g, respectively, with a statistically significant difference (p = 0.040).

**Table 1:** Baseline characteristics of participants based on serum AGP concentrations.

	Serum AGP concentrations (mg/dl)			P-value
	Group 1	Group 2	Group 3	
	<63 mg/dl (n=855)	63-84 mg/dl (n=864)	>84 mg/dl (n=860)	
**Age**	29.98 ± 9.85	31.90 ± 10.19	32.90 ± 10.24	**<0.001**
**Race**				**<0.001**
Mexican American	136 (15.91%)	189 (21.88%)	163 (18.95%)	
Other Hispanic	80 (9.36%)	120 (13.89%)	80 (9.30%)	
Non-Hispanic White	259 (30.29%)	231 (26.74%)	329 (38.26%)	
Non-Hispanic Black	155 (18.13%)	175 (20.25%)	192 (22.33%)	
Other/multiracial	225 (26.32%)	149 (17.25%)	96 (11.16%)	
**BMI (kg/m^2^)**	24.93 ± 5.46	28.99 ± 6.94	33.90 ± 8.57	**<0.001**
**WC (cm)**	85.02 ± 13.24	93.92 ± 15.49	105.63 ± 18.36	**<0.001**
**Hypertension**				**<0.001**
Yes	67 (7.84%)	116 (13.43%)	164 (19.07%)	
No	788 (92.16%)	748 (86.57%)	696 (80.93%)	
**Hypercholesterolemia**				**0.014**
Yes	94 (10.99%)	110 (12.73%)	135 (15.70%)	
No	761 (89.01%)	754 (87.27%)	725 (84.30%)	
**Diabetes**				**<0.001**
Yes	19 (2.22%)	42 (4.86%)	57 (6.63%)	
No	824 (96.37%)	808 (93.52%)	786 (91.40%)	
Borderline	12 (1.40%)	14 (1.62%)	17 (1.98%)	
**Sleep Disorders**				**<0.001**
Yes	151 (17.66%)	181 (20.95%)	273 (31.74%)	
No	704 (82.34%)	683 (79.05%)	587 (68.26%)	
**UACR (mg/g)**	18.03 ± 82.63	38.21 ± 301.63	57.35 ± 737.85	**0.040**

Mean ± SD for continuous, N (%) for categorical. Kruskal Wallis rank sum test for continuous variables, Fisher's exact probability test for categorical variables with theoretical number < 10. Bold values indicate p-value < 0.05. AGP, Alpha-1-acid glycoprotein; UACR, urine albumin-to-creatinine ratio; BMI, body mass index; WC, waist circumference.

### Correlation between serum AGP concentration and UACR

In this study, we designed three Cox regression models to investigate the correlation between serum AGP concentration and UACR (**Table 2**). After adjusting for variables in the three models, the multivariate adjusted effect values βs and their 95% confidence intervals (95% CIs) were 1.45 (0.70, 2.19) (p=0.0001), 1.50 (0.74, 2.26) (p=0.0001), and 1.89 (1.02, 2.76) (p < 0.0001) for model 1, 2, and 3, respectively. Considering each group, compared to group 1, in model 1, the βs for group 2 and 3 were 20.17 (-23.59, 63.94) and 39.31 (-4.51, 83.13); in model 2, the βs for groups 2 and 3 were 19.82 (-24.41, 64.05) and 39.22 (-5.51, 83.95); in model 3, the βs for groups 2 and 3 were 23.78 (-21.39, 68.94) and 47.41 (-2.65, 97.47). Yet, the differences between these βs were not statistically significant. As shown in **Supplementary Tables S1**–**S3**, no significant differences were found between the sensitivity analyses and the results after correcting for multiple testing using the Benjamini-Hochberg procedure and the previous results, which enhances the robustness of our results.

**Table 2:** βs (95% CIs) for association between serum AGP concentrations and UACR.

	Model 1		Model 2		Model 3	
	β (95% CI)	p-value	β (95% CI)	p-value	β (95% CI)	p-value
**Serum AGP concentration (mg/dl)**	1.45 (0.70, 2.19)	**0.0001**	1.50 (0.74, 2.26)	**0.0001**	1.89 (1.02, 2.76)	**<0.0001**
**Group 1**	ref	ref	ref	ref	ref	ref
**Group 2**	20.17 (-23.59, 63.94)	0.3664	19.82 (-24.41, 64.05)	0.3799	23.78 (-21.39, 68.94)	0.3023
**Group 3**	39.31 (-4.51, 83.13)	0.0788	39.22 (-5.51, 83.95)	0.0858	47.41 (-2.65, 97.47)	0.0635

Model 1 adjust for: none.

Model 2 adjust for: age; race.

Model 3 adjust for: age, race, BMI, waist circumference, history of hypertension, and hypercholesterolemia, diabetes, sleep disorders.

AGP, Alpha-1-acid glycoprotein; UACR, urine albumin-to-creatinine ratio; CI, confidence interval.

### Exploration of nonlinear correlation

**Figure 2:**
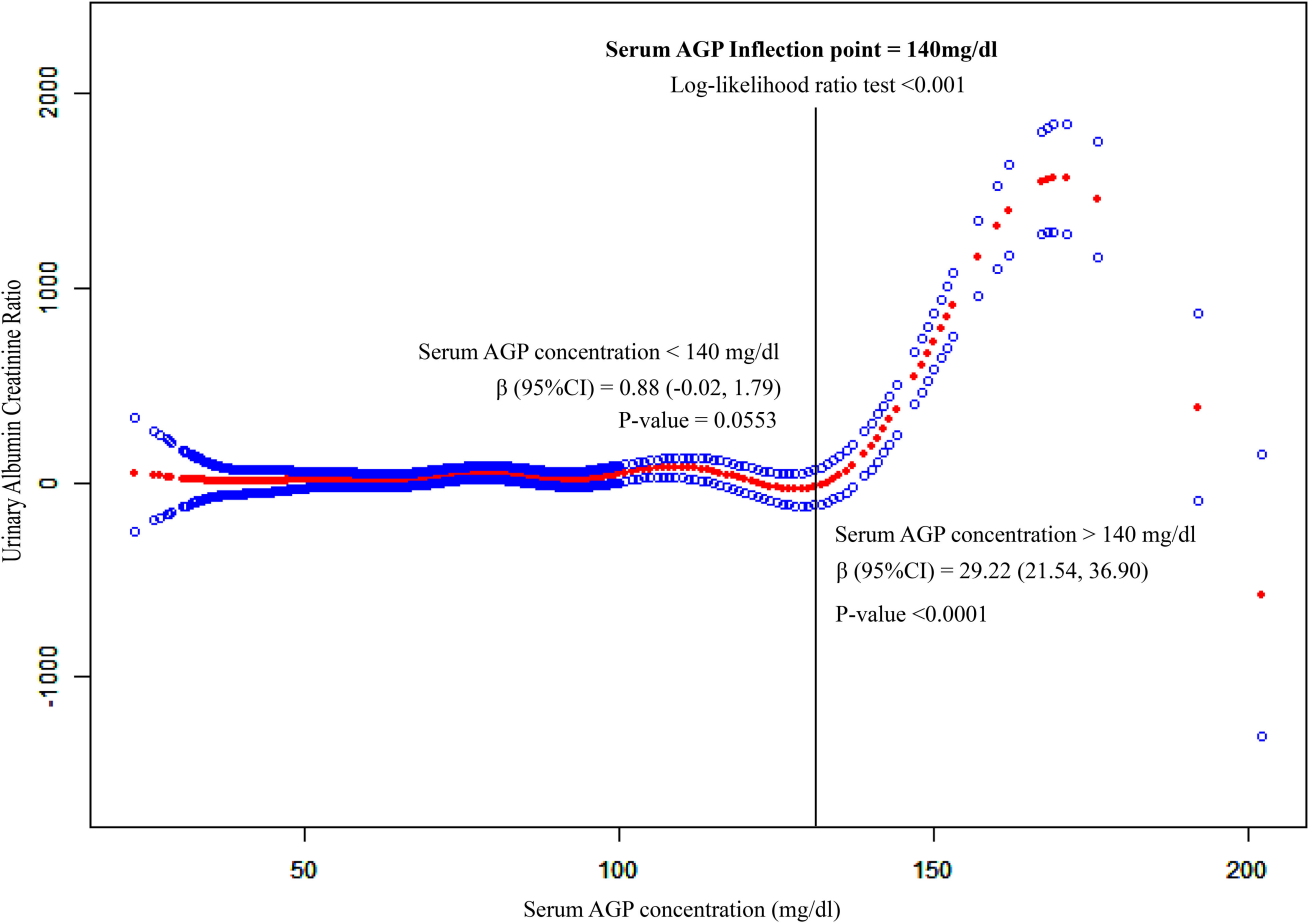
Non-liner association between serum AGP concentrations and UACR. This model adjusted for age, race, BMI, and waist circumference, and history of hypertension, hypercholesterolemia, diabetes and sleep disorders. The red and blue lines represent the estimated effect value and its corresponding 95% CI, respectively. S.AGP.C expresses serum Alpha-1-Acid Glycoprotein concentration. Its unit is mg/dl. UACR expresses the ratio of the albumin concentration in urine to the corresponding creatinine concentration.

To explore non-linear correlation by employing smooth curve fitting (penalized spline method), notably, we observed that serum AGP concentration exhibited an L-shaped correlation with UACR in **Figure 2**. Furthermore, we integrated Cox proportional risk regression models and two-piecewise linear regression model for threshold effect analysis (Log-likelihood ratio test<0.001). We discovered that the inflection point was 140 mg/dl for serum AGP (**Table 3**). When serum AGP concentration was less than 140 mg/dL, the UACR was plateaued with a β (95% CI) of 0.88 (-0.02, 1.79) (p = 0.0553). When the serum AGP concentration was higher than 140 mg/dl, there was a significant and substantial increase in the UACR with a β (95% CI) of 29.22 (21.54, 36.90) (p < 0.0001).

**Table 3:** Threshold effect analysis of serum AGP concentrations on UACR.

Fitting by the two-piecewise linear regression model	β (95%CI)	P-value
Serum AGP Inflection point (140mg/dl)		
Serum AGP concentration < 140 mg/dl	0.88 (-0.02, 1.79)	0.0553
Serum AGP concentration > 140 mg/dl	29.22 (21.54, 36.90)	**<0.0001**
Log-likelihood ratio test		**<0.001**

The covariates employed in this model were identical to those depicted in **Figure 2**. Bold values indicate p-value < 0.05.

AGP, Alpha-1-acid glycoprotein; UACR, urine albumin-to-creatinine ratio. CI, confidence interval.

### Stratified analyses

**Figure 3:**
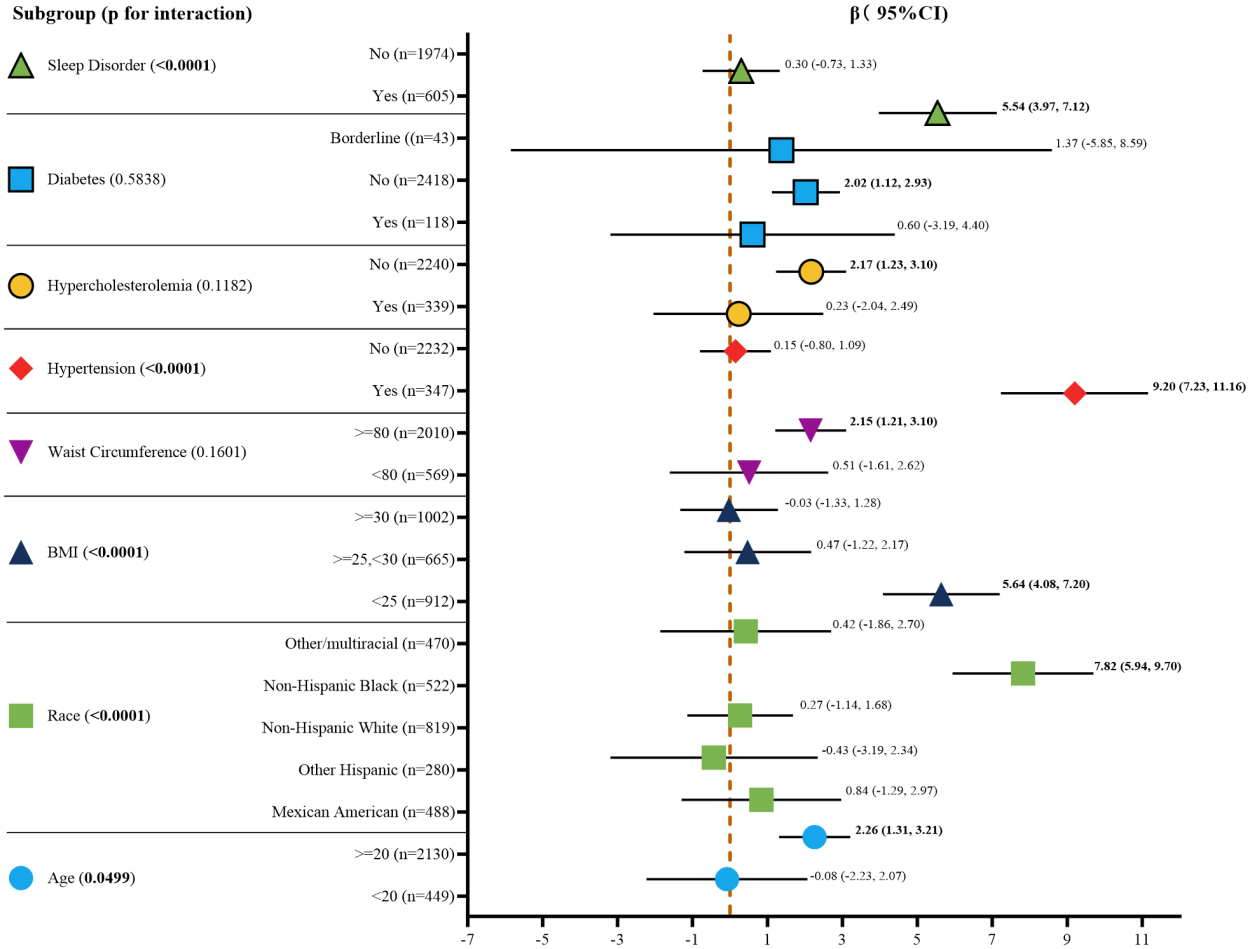
Subgroup analysis for the association between serum AGP concentrations and UACR. Age, race, BMI, waist circumference, and history of hypertension, and hypercholesterolemia, diabetes, sleep disorders were all adjusted except for the covariates themselves. Bold values indicate p-value < 0.05, which is statistically significant. BMI, body mass index; CI, confidence interval.

The results of the stratified analysis, after adjusting for all covariates, are depicted in **Figure 3**. The impact of serum AGP on UACR varied significantly across each subgroup of age, race, BMI, waist circumference, history of hypertension, hypercholesterolemia, diabetes, and sleep disorders. Interaction tests suggested potential effect modifications by age, race, BMI, hypertension, and sleep disorders, with p-values for interaction of 0.0499, <0.0001, <0.0001, <0.0001, and <0.0001, respectively. However, further studies are needed to confirm these findings and explore the underlying mechanisms.

## Discussion

The primary motivation of this study was to explore the correlation between serum AGP concentration and UACR, as this association has not been quantitatively investigated in prior research. Understanding this relationship is critical for improving the assessment of kidney function and identifying potential biomarkers for early detection and management of kidney disease.

The present study illustrated an L-shaped correlation between serum AGP level and UACR, and a serum AGP level >140 mg/dl was associated with a dramatic increase in UACR, which indicates deteriorating renal function. Our findings agree with the existing literature, which recognizes AGP as a potential inflammatory marker that is associated with renal damage. However, unlike previous studies that had been restricted to qualitative correlations, our research provides a quantitative analysis, offering clinicians a more precise tool with which to measure kidney health. Not only does this enhance the understanding of AGP’s role in kidney disease, but it also underscores its potential utility in clinical practice for risk stratification and treatment planning.

In this study, participants were equally divided into groups with high, medium, and low concentrations based on serum AGP concentrations. Participants with high serum AGP concentrations exhibited higher age, BMI, and waist circumference. This could indicate a more severe inflammatory condition present in older and obese individuals. Longling Wang’s study is also consistent with our findings. Wang suggested that age and obesity cause senescence of immune cells, which results in the body remaining in a state of chronic inflammation (25). Since 449 child participants were included in this study, we also are in agreement with Anna Medyńska’s study, which demonstrated that α1-AGP was significantly higher in obese children than in healthy controls (13). Nevertheless, participants with higher serum AGP concentrations were less likely to have hypertension, diabetes, and sleep disorders. This probably indicates that hypertension, diabetes, and sleep disorders have a greater impact on the liver and are more detrimental to serum AGP production. This is clearly contrary to our current knowledge. Unfortunately, no studies have been conducted to propose a theoretical mechanism for this. In Cox regression analysis, the β between serum AGP concentration and UACR increased significantly with the addition of variables. It suggests that the covariates added in the different models are prominent meaningful. Similar to the present study, Peters’s study showed that urinary AGP 1 was positively associated with diabetes duration, ACR, urea, creatinine, triglycerides, cholesterol, and LDL. While urinary AGP 1 was negatively associated with eGFR, albumin, and HDL (20). This may also explain why obese individuals have higher serum AGP concentrations. This could also explain, on the other hand, why obese individuals have higher serum AGP concentrations. Also compared to Peters’s study (n=90), this study (n=2579) included a larger number of participants, making the results more informative.

In our analysis, covariates were selected based on their established relevance to both AGP and UACR. Age, sex, BMI, and renal function markers (e.g., serum creatinine, eGFR) were included, as they influence inflammatory markers like AGP and kidney function indicators such as UACR (21). AGP is elevated in individuals with cardiovascular disease, inflammation, and metabolic disorders, conditions that also affect renal function (26, 27). While our analysis adjusted for key covariates, certain factors—such as genetic predispositions, dietary patterns, and unmeasured lifestyle variables—were not accounted for. Genetic polymorphisms in inflammatory pathways can influence AGP levels, and dietary factors (e.g., protein or fat intake) may impact both AGP and UACR (28). Additionally, socioeconomic status, physical activity, and medication use (e.g., statins, antihypertensives) could act as confounders. Statins have been shown to reduce proteinuria in some populations (28–30), while antihypertensives may affect AGP and UACR through their anti-inflammatory and blood pressure-lowering effects (31). Given these limitations, our results should be interpreted with caution. Future studies should incorporate these additional covariates to strengthen the validity of the observed associations and provide deeper insights into the relationship between AGP and kidney function.

The L-shaped correlation between serum AGP and UACR was found by this study. As can be seen in **Figure 2**, when the serum AGP concentration was lower than 140 mg/dl, the UACR remained in a steady pattern, indicating that there was no significant change in the ability of the kidney to filter albumin and creatinine. When the serum AGP concentration was higher than 140 mg/dL, the UACR increased significantly in a linear form, suggesting that 140 mg/dL may be the limit of the kidney compensation for the acute time-phase protein AGP. This concentration may be a meaningful boundary for us to emphasize when using serum AGP for kidney function monitoring. Huabin Wang ‘s study found that the critical concentration of urinary orosomucoid (ORM) 1 protein for the early diagnosis of renal impairment is 2.53 mg/L (16), and the urinary ORM1-to- creatinine ratio (>3.69 mg/g) has the high diagnostic efficiency for the early screening of renal function. The urinary ORM1-to-creatinine ratio (>3.69 mg/g) has the high diagnostic efficiency for the early screening of renal impairment in type-2 diabetes patients.

AGP is an acute-phase response protein primarily synthesized by the liver. Its expression significantly increases in response to inflammation, infection, or tissue injury. With anti-inflammatory, immunomodulatory, and drug-binding properties, AGP may play a protective role in kidney injury by modulating inflammatory responses, reducing oxidative stress, and inhibiting apoptosis (8, 9). In both acute kidney injury and chronic kidney disease, AGP levels can fluctuate, highlighting its potential as a biomarker for early detection and disease monitoring. AGP complements existing clinical markers such as UACR and eGFR. While UACR and eGFR primarily indicate current kidney damage or dysfunction, AGP reflects ongoing inflammatory processes, providing additional insights into disease progression (32). Studies have shown that AGP is independently associated with renal outcomes, even after adjusting for traditional CKD markers, underscoring its utility as a complementary biomarker (25). Integrating AGP into routine clinical testing alongside UACR and eGFR could improve CKD risk stratification, enable earlier therapeutic interventions, and potentially slow disease progression (25, 33). The role of AGP in kidney disease parallels that of other inflammatory markers, such as high-sensitivity C-reactive protein (hs-CRP), neutrophil-to-lymphocyte ratio (NLR), and monocyte chemoattractant protein-1 (MCP-1). For instance, Aljazi’s study demonstrated that hs-CRP and dietary quality (DQ) were significantly negatively correlated with eGFR, regardless of baseline eGFR, suggesting their potential to delay kidney disease progression (3). Similarly, elevated serum AGP levels (above 140 mg/dl) were associated with increased UACR, indicating a possible contributing factor to kidney disease. Additionally, AGP is upregulated in the synovial fluid and synovium of rheumatoid arthritis patients, suggesting a similar pro-inflammatory role in disease development (12).

While our study has a particular focus on women, this is because after excluding all the missing key exposure variables, outcome variables and covariates, the only participants left in this study were females. However, we recognize the importance of considering the broader implications for men and other populations. Studies have shown that both AGP levels and UACR can vary by gender. For example, some studies have reported higher baseline AGP levels in men, which may be due to differences in inflammatory responses or hormonal influences (34–36). Similarly, UACR levels may vary by gender, with males typically having lower UACR values than females in certain age groups, which may affect the generalizability of our findings (37, 38). Although our analyses were limited to female participants, future studies should explore whether the L-shaped association between AGP and UACR persists across gender or other population groups. Such an investigation could increase the translational value of our findings and be consistent with best practices to ensure broader applicability to diverse populations. We also encourage additional studies focused on understanding sex-specific factors that may modify the relationship between AGP and renal prognosis.

## Strengths and limitations

This study offers several notable advantages. Firstly, it utilizes a nationally representative sample from the U.S., encompassing both serum AGP and UACR measurements. The substantial sample size enhances the generalizability of our findings. Secondly, by meticulously adjusting for a range of demographic variables, physical measurements, disease histories, and other potential confounders, we have fortified the validity of our conclusions. Thirdly, the serum AGP concentrations in the NHANES database were measured using rigorous, standardized methodologies, ensuring the robustness of our data analysis. Lastly, our stratified analyses underscore the robustness of the observed relationship between serum AGP and UACR across varied demographic and clinical subgroups. However, this study is not without limitations. Despite accounting for numerous covariates, we could not completely eliminate the influence of all potential confounders, such as genetic and dietary factors. Additionally, the study’s design and the availability of data precluded the inclusion of male participants, thereby limiting the scope of our findings.

## Conclusion

This study was the first to demonstrate a correlation between serum AGP and UACR. It also revealed an L-shaped correlation with a threshold of 140 mg/dL. These findings indicate that suggesting that serum alpha-1-acid glycoprotein may be a potential target for future attention to renal status of females, but needs to be confirmed in clinical trials.

## Data Availability

Publicly available datasets were analyzed in this study. This data can be found here: https://www.cdc.gov/nchs/nhanes/index.htm.
