# Supplementary material for: L-shaped correlation between serum alpha-1-acid glycoprotein concentration and urinary albumin creatinine ratio in females: a cross-sectional survey
Source: Front Endocrinol (Lausanne). 2025 Mar 24;16:1438695. doi: 10.3389/fendo.2025.1438695 (PMC11973102; doi:10.3389/fendo.2025.1438695)
Supplement: Supplementary file 1 [file Table1.docx]

**Supplementary Files**

Table S1. βs (95% CIs) for correlation between serum AGP concentrations and UACR excluding participants were diagnosed with diabetes within the first two years of follow-up.

Table S2. βs (95% CIs) for correlation between serum AGP concentrations and UACR excluding participants who had self-reported CKD.

Table S3. βs (95% CIs) for correlation between serum AGP concentrations and UACR using Benjamini-Hochberg procedure.

| **Table S1. βs (95% CIs) for correlation between serum AGP concentrations and UACR excluding participants were diagnosed with diabetes within the first two years of follow-up.** | | | | | | |
| --- | --- | --- | --- | --- | --- | --- |
|  | Model 1 | | Model 2 | | Model 3 | |
|  | β (95% CI) | p-value | β (95% CI) | p-value | β (95% CI) | p-value |
| Serum AGP concentration (mg/dl) | 1.55 (0.76, 2.06) | **0.0001** | 1.67 (0.86, 2.33) | **0.0001** | 1.85 (1.03, 2.59) | **<0.0001** |
| Group 1 | ref | ref | ref | ref | ref | ref |
| Group 2 | 18.98 (-21.35, 58.02) | 0.2588 | 20.37 (-21.54, 72.45) | 0.4216 | 24.63 (-20.27, 69.75) | 0.3351 |
| Group 3 | 40.26 (-3.88, 79.23) | 0.0647 | 40.33 (-4.45, 82.98) | 0.0774 | 49.65 (-2.24, 98.16) | 0.0553 |
| *Model 1 adjust for: none. | | | | | | |
| *Model 2 adjust for: age; race. | | | | | | |
| *Model 3 adjust for: age, race, BMI, waist circumference, history of hypertension, and hypercholesterolemia, diabetes, sleep disorders.  *AGP: Alpha-1-acid glycoprotein; UACR: urine albumin-to-creatinine ratio; CI: confidence interval. | | | | | | |

| **Table S2. βs (95% CIs) for correlation between serum AGP concentrations and UACR excluding participants who had self-reported CKD.** | | | | | | |
| --- | --- | --- | --- | --- | --- | --- |
|  | Model 1 | | Model 2 | | Model 3 | |
|  | β (95% CI) | p-value | β (95% CI) | p-value | β (95% CI) | p-value |
| Serum AGP concentration (mg/dl) | 1.38 (0.69, 1.95) | **<0.0001** | 1.83 (0.91, 2.59) | **0.0001** | 1.91 (1.14, 2.79) | **<0.0001** |
| Group 1 | ref | ref | ref | ref | ref | ref |
| Group 2 | 19.38 (-20.52, 60.09) | 0.4662 | 21.32 (-19.65, 76.95) | 0.6924 | 23.59 (-18.75, 73.39) | 0.5276 |
| Group 3 | 41.36 (-2.18, 80.23) | 0.1254 | 42.53 (-4.02, 86.63) | 0.0557 | 48.14 (-2.59, 96.34) | 0.0769 |
| *Model 1 adjust for: none. | | | | | | |
| *Model 2 adjust for: age; race. | | | | | | |
| *Model 3 adjust for: age, race, BMI, waist circumference, history of hypertension, and hypercholesterolemia, diabetes, sleep disorders.  *AGP: Alpha-1-acid glycoprotein; UACR: urine albumin-to-creatinine ratio; CI: confidence interval. | | | | | | |

| **Table S3. βs (95% CIs) for correlation between serum AGP concentrations and UACR using Benjamini-Hochberg procedure.** | | | | | | |
| --- | --- | --- | --- | --- | --- | --- |
|  | Model 1 | | Model 2 | | Model 3 | |
|  | β (95% CI) | p-value* | β (95% CI) | p-value* | β (95% CI) | p-value* |
| Serum AGP concentration (mg/dl) | 1.45 (0.70, 2.19) | <0.0001 | 1.50 (0.74, 2.26) | <0.0001 | 1.89 (1.02, 2.76) | <0.0001 |
| Group 1 | ref | ref | ref | ref | ref | ref |
| Group 2 | 20.17 (-23.59, 63.94) | 0.2443 | 19.82 (-24.41, 64.05) | 0.2533 | 23.78 (-21.39, 68.94) | 0.2015 |
| Group 3 | 39.31 (-4.51, 83.13) | 0.0788 | 39.22 (-5.51, 83.95) | 0.0858 | 47.41 (-2.65, 97.47) | 0.0635 |
| *Model 1 adjust for: none. | | | | | | |
| *Model 2 adjust for: age; race. | | | | | | |
| Model 3 adjust for: age, race, BMI, waist circumference, history of hypertension, and hypercholesterolemia, diabetes, sleep disorders.  *AGP: Alpha-1-acid glycoprotein; UACR: urine albumin-to-creatinine ratio; CI: confidence interval.  * The Benjamini-Hochberg procedure was implemented to adjust for multiple testing. | | | | | | |
